# Analyses of effect factors associated with the postoperative dissatisfaction of patients undergoing open-door laminoplasty for cervical OPLL: a retrospective cohort study

**DOI:** 10.1186/s13018-019-1208-8

**Published:** 2019-05-28

**Authors:** Sen Liu, Si-Dong Yang, Xi-Wen Fan, Da-Long Yang, Lei Ma, Jia-Yuan Sun, Wen-Yuan Ding

**Affiliations:** 1 Department of Spinal Surgery, The Third Hospital of Hebei Medical University, 139 Ziqiang Road, Shijiazhuang, 050051 People’s Republic of China; 2 Hebei Provincial Key Laboratory of Orthopaedic Biomechanics, 139 Ziqiang Road, Shijiazhuang, 050051 People’s Republic of China

**Keywords:** Effect factors, Dissatisfaction, OPLL, Complication, Hospitalization cost, Neck pain, Symptom recurrence

## Abstract

**Objectives:**

This study aimed to investigate the effect factors associated with the postoperative dissatisfaction of patients undergoing open-door laminoplasty for cervical OPLL.

**Methods:**

In this study, 194 patients, who underwent open-door laminoplasty for cervical OPLL from January 2009 to January 2016, were retrospectively reviewed. The Patient Satisfaction Index (PSI) was collected at discharge, 6 months, 1 year, and the last follow-up. According to the PSI, patients were divided into satisfied group and dissatisfied group. The possible effect factors included demographic variables and surgery-related variables.

**Results:**

At discharge, 42 (21.6%) patients were in the dissatisfied group, as compared to the satisfied group, the hospitalization cost, hospital stay, postoperative depression, the axial neck pain, delayed wound healing, and VAS-neck had significant statistical differences. At 6-month follow-up, 25 (12.9%) patients were in the dissatisfied group. The axial neck pain and JOA score had significant statistical differences between the two groups, and no significant differences were found between the two groups in other items. At 1 year with 18 (9.3%) dissatisfied patients and last follow-up with 14 (7.2%) dissatisfied patients, the JOA score and symptom recurrence had significant statistical differences. For further analysis, the dissatisfied patients with axial neck pain at 6 months were significantly higher than that at other terms and the JOA score of the two groups increased gradually with prolonging of restoration years but compared with the dissatisfied group, the JOA scores were obviously better in the satisfied group at the last follow-up.

**Conclusions:**

Overall, to patients undergoing open-door laminoplasty for cervical OPLL, hospitalization cost and neck pain might be mainly associated with patient dissatisfaction at the early and middle recovery. Patient dissatisfaction at the long-term treatment outcome might be mainly associated with the low improvement rate of JOA score and symptom recurrence.

## Introduction

**Fig. 1:**
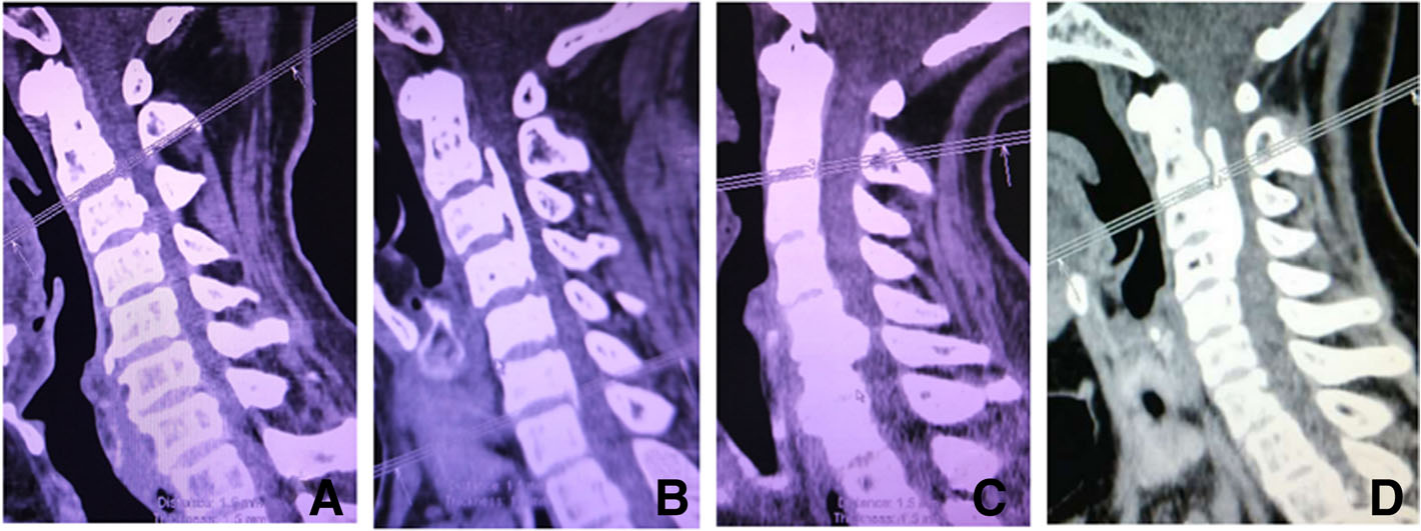
**a** Segmental type. **b** Mixed type. **c** Focal type. **d** Continuous type

Ossification of the posterior longitudinal ligament (OPLL) is a heterotropic ossification that occupies the spinal canal and causes myelopathy. The OPLL can be divided into four types: segmental type, mixed type, focal type, and continuous type (Fig. 1). OPLL is a common cause of myelopathy in patients older than 55 years of age [1, 2]. More than that, OPLL can also lead to defecation dysfunction even paralysis if accompanied by cervical cord injury. For cases with cervical myelopathy due to OPLL, surgery treatment, including anterior cervical corpectomy and fusion (ACCF), anterior cervical discectomy and fusion (ACDF), laminoplasty (LP), and laminectomy arthrodesis with or without instrumentation, can provide direct ossification resection or indirect decompression [3–5]. Anterior surgery is generally considered appropriate for the cases with the ossification range within three vertebral bodies, the thickness less than 5 mm, and the stenosis rate less than 45%. Due to technically demanding and higher potential risk of complications such as nonunion, dislodgment of grafts, dural tears and neurological deterioration of anterior surgery, laminoplasty is still the most widely used procedure in the treatment of C-OPLL.

Many previous studies have shown satisfactory outcomes of the surgical procedure for the treatment of OPLL, but there were still some dissatisfied cases. The characteristics of the satisfied and dissatisfied patients are different in some ways. Satisfied patients are more willing to cooperate with their healthcare providers by providing important medical information and continue using medical care services. They usually obtained good results because of less pre- and postoperative risk factors. On the contrary, the dissatisfied patients may make treatment less effective, either by neglecting to seek care when needed or refusing to comply with the prescribed course of treatment [6, 7]. Since the dissatisfaction of the patient can adversely affect the evaluation of the effectiveness of the treatment, it is necessary for medical providers to provide the right and effective treatment to strive to satisfy their patients undergoing spine surgery.

## Materials and methods

### Inclusion of patients

Between January 2009 and January 2016, a total of 194 consecutive patients (98 men and 96 women) who were diagnosed with cervical OPLL and needed operative managements were examined prospectively. Ethics Committee of The Third Hospital of Hebei Medical University approved the study, and written informed consents were obtained from all patients before they were recorded. The inclusion criteria were the following: continuous, mixed, isolated, and localized ossification presence of cervical spinal stenosis with neurological dysfunctions; patients treated with open-door laminoplasty, inefficacious with conservative treatment; complete imaging support for OPLL. The exclusion criteria were the following: the presence of infection, trauma; cervical ossification of the ligamenta flava; association with thoracic or lumbar diseases; spine deformity; previous history of spinal surgery; any concurrent spinal tumors; medical causes of non-compressive myelopathy; unwillingness to participate in the study.

### Study variables

The possible predicting factors include three parts: demographic variables, surgical-related variables, and complications. The following are the demographic variables collected at baseline: age, sex, BMI, smoking, drinking, heart disease, hypertension, and diabetes. And the surgical-related variables include course of disease, lesions vertebral, spinal canal stenosis rate, hospitalization cost, course of disease, operation time, blood loss, hospital stay, length of the incision, presence of intramedullary high-signal intensity, adhesion to the dural mater, preoperative and postoperative neck VAS and JOA scores, and complications. The patients were followed up 2 years after open-door laminoplasty for the long-term clinical evaluation, and short-and long-dated complications were respectively collected. The satisfaction of the patients was collected at the last follow-up evaluation. We choose the 2-year follow-up interval at which time we wish outcomes were expected to be optimal to assess the satisfaction of the patients.

### Hospitalization cost evaluation

The hospitalization costs were collected at the last follow-up, including drug expense, check expense, operation expense, nursing expenses on the patient’s total expense list when discharged, and postoperative rehabilitation costs. Hospitalization expenses may indirectly reflect the severity of the patient’s condition and the extent of the impact on the family.

### Treatment method

All the patients underwent open-door laminoplasty for cervical OPLL. The patients were placed in the prone position while under general anesthesia. Through bilateral paraspinal muscle dissection and the supraspinous ligaments entirely preserved, the interspinous ligaments were cut at the superior and inferior ends of the C3–6 or C3–7 levels. A full-thickness trough was drilled at the junction of the lateral mass and the lamina with a high-speed burr on the right side as the open-door, and a partial-thickness trough was drilled on the left side as the door-hinge. The lamina was elevated from the open-door toward the hinge side for approximately 8 to 10 mm and stabilized with 8 or 10 mm mini-plates and screws. A drainage tube was routinely placed after the operation. The mecobalamin was used for promoting neurological recovery after the operation.

### Satisfaction evaluation

In each patient, operative procedure and collection of study variables were completed by different personnel to avoid biased response. The patient was provided the first questionnaire about the demographic dates before operation. The Patient Satisfaction Index (PSI) [8] was used to describe self-evaluation of the outcome and response of 1 or 2 was considered to indicate a satisfying outcome and 3 or 4 to indicate a dissatisfied outcome (Table 1). According to the PSI, patients were divided into satisfied group and dissatisfied group and the Patient Satisfaction Index (PSI) was collected at discharge, 6 months, 1 year, and last follow-up by the researchers over the telephone questionnaire associating with patient satisfaction and functioning. The visual analog scale (VAS) is a sensitive and reliable clinical procedure for the assessment of pain degree, which consists of a horizontal line 100 mm in length. The ends of the horizontal line point “0” and “100,” respectively representing “no pain” and “worst imaginable pain.” The middle section shows different degrees of pain [9]. Japanese Orthopaedic Association Scores (JOA) is mainly used to evaluate the functional disorder of the human body and JOA improvement rate is considered to describe the function improvement after surgery, with the result of 100% considered to be all healed, 60–100% to be remarkable, 25–60% to be effective, less than 25% to be generally ineffective compared with preoperative values. The cognitive level of the disease, we believe is related to the following aspects: household income, education level, occupation, household register, which are the process of deep understanding of the disease. Whether there is income stress for family is considered to be judged according to income stability, health insurance, pensions, and big trouble to the family. According to their occupation, patients were divided into mental workers, with small physical strength labor, such as executive, company employee, teacher, government official, civil servant, designer, and engineer, and manual workers, with amount of physical strength labor, such as miner, driver, construction worker, and farmer.

**Table 1:** Patient Satisfaction Index (PSI)

PSI	Patient responses
1	Surgery met my expectations
2	Surgery improved my condition enough so that I would go through it again for the same outcome
3	Surgery helped me but I would not go through it again for the same outcome
4	I am the same or worse compared to before surgery

*PSI* patient satisfaction index

### Statistical analysis

Comparative analysis with patient dissatisfaction as the dependent variable was done by independent samples *t* tests and chi-square. Age, body mass index (BMI), course of disease, spinal canal stenosis rate, preoperative VAS-neck and JOA, postoperative neck VAS and JOA, hospitalization cost were analyzed by independent samples *t* tests, and sex, smoking, drinking, heart disease, hypertension, diabetes, education level, occupation, household register, type of ossification, spinal canal stenosis rate, preoperative kyphosis, K-line (−), presence of intramedullary high-signal intensity, lesions vertebral, presence of intramedullary high-signal intensity, adhesion to the dura mater, early, and long-dated complications were analyzed by chi-square. The statistical significant value was set at *P* < 0.05 in the univariate analyses. All statistical analyses were carried out by SPSS software version 13.0 (SPSS, Inc., Chicago, IL, USA) (Table 2).

**Table 2:** The main demographic variables in the satisfied and dissatisfied patients at discharge

	Dissatisfaction (*n* = 42)	Satisfaction (*n* = 152)	*P* value
Age (years)	56.8 ± 8.8	56.2 ± 9.8	0.703
Sex (male/female)	20/22	78/74	0.671
BMI (kg/m^2^)	24.5 ± 2.9	24.2 ± 3.1	0.627
Smoking (yes/no)	13/29	56/96	0.480
Drink (yes/no)	17/25	66/86	0.733
Heart disease (yes/no)	13/29	30/122	0.121
Hypertension (yes/no)	18/24	46/106	0.124
Diabetes (yes/no)	16/26	40/112	0.147

The main demographic variables in the two groups were not significantly different at discharge (*P* > 0.05)

## Results

On the day before surgery, 194 patients (98 men and 96 women) were registered on the books for evaluation voluntarily. The cohort of patients was integrated before discharge. At the time of the 2-year follow-up, no patient was lost to follow-up.

At discharge, 42 (21.6%) patients were in dissatisfied group, as compared to satisfied group, the hospitalization cost, hospital stay, postoperative depression, the axial neck pain, delayed wound healing, and VAS-neck had significant statistical differences (Table 3). However, no significant differences were found between the two groups in other items (Table 2).

**Table 3:** The related risk factors of satisfied and dissatisfied patients at discharge

	Dissatisfaction (*n* = 42, 21.6%)	Satisfaction (*n* = 152, 78.4%)	*P* value
Course of disease (months)	14.06 ± 2.36	13.85 ± 2.01	0.577
Operation time (min)	155.6 ± 18.8	156.2 ± 25.6	0.888
Blood loss (ml)	460.6 ± 58.1	473 ± 54.5	0.198
Hospital stay (days)	12.1 ± 1.8	10.9 ± 1.9	0.001*
Length of the incision (cm)	15.2 ± 0.9	15.4 ± 1.1	0.492
Hospitalization cost (thousand RMB)	39.8 ± 8.7	37.1 ± 7.6	0.045
Lesions vertebral (C3–6/C3–7)	16/26	65/87	0.581
Spinal canal stenosis rate	49.2 ± 7.0	51.5 ± 7.7	0.083
Preoperative neck VAS	4.45 ± 0.89	4.37 ± 0.84	0.573
Preoperative JOA scores	7.42 ± 0.89	7.56 ± 0.79	0.325
Postoperative neck VAS	4.19 ± 1.35	3.09 ± 1.17	< 0.001**
Postoperative JOA scores	9.48 ± 1.02	9.54 ± 0.78	0.615
Complications			
Wound infected (yes/no)	5/37	9/143	0.185
Dysphagia (yes/no)	2/40	4/148	0.480
C5 paralysis (yes/no)	6/36	10/142	0.108
Axial pain (yes/no)	20/22	43/109	0.018*
Cerebrospinal fluid leakage (yes/no)	9/33	18/134	0.112
Hematoma (yes/no)	5/37	11/141	0.330
Delayed wound healing (yes/no)	16/26	23/129	< 0.001**
Postoperative depression (yes/no)	14/28	20/132	< 0.001**
Others (yes/no)	6/36	15/137	0.415

*The difference possessing statistical significance

*P < 0.05, **P = 0.000

**Fig. 2:**
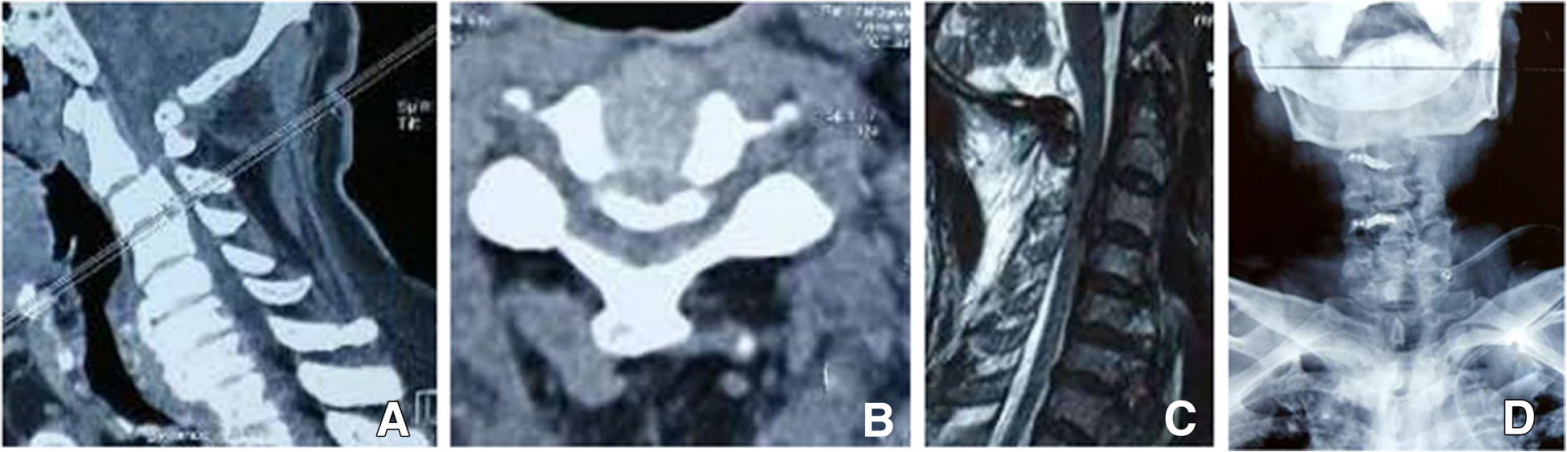
A 60-year-old male patient (**a**, **b**, **c**, and **d**) of the dissatisfied group at 6-month and 1-year follow-up developed numbness and weakness in his four extremities for 1 year, together with unbalance gait for 3 months. After the operation, his JOA scores improved from 9.7 pre-operation to 14.6 post-operation but he suffered from severe axial pain for 9 months which seriously affected the quality of life

At 6-month follow-up, 25 (12.9%) patients were in dissatisfied group. The axial neck pain, postoperative depression, and JOA score had significant statistical differences between the two groups (Table 4). Further analysis, the dissatisfied patients with axial neck pain at 6 months was significantly higher than that at other terms (Fig. 2). No significant differences were found between the two groups in other items.

**Table 4:** The related risk factors of satisfied and dissatisfied patients at the 6-month follow-up

	Dissatisfaction (*n* = 25, 12.9%)	Satisfaction (*n* = 169, 87.1%)	*P* value
Hospitalization cost (thousand RMB)	3.91 ± 0.89	3.74 ± 0.77	0.334
JOA scores after 6 months	10.89 ± 1.28	11.58 ± 1.45	0.006*
VAS neck after 6 months	3.26 ± 1.08	2.01 ± 1.21	< 0.001**
Complications			
Axial pain (yes/no)	17/8	24/145	< 0.001**
Delayed wound healing (yes/no)	7/18	32/137	0.291
Postoperative depression (yes/no)	4/21	9/160	0.045*
Symptom recurrence (yes/no)	1/24	2/167	0.379

*The difference possessing statistical significance

*P < 0.05, **P = 0.000

**Fig. 3:**
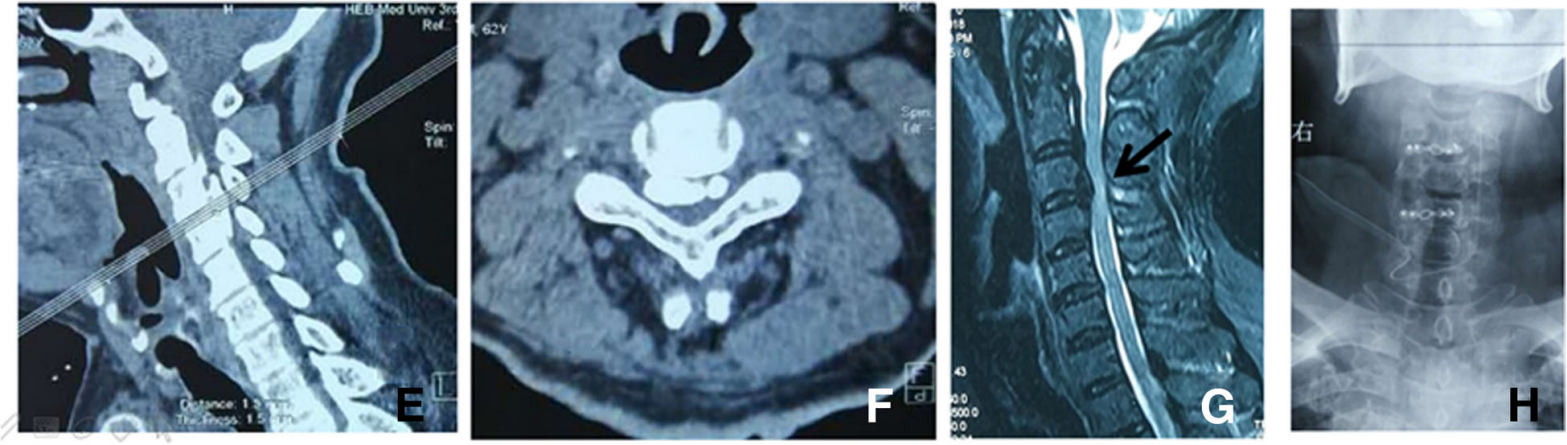
A 53-year-old male patient (**e**, **f**, **g**, and **h**) of the dissatisfied group at the last follow-up developed numbness in his two hands and weakness in his four extremities for 3 years. He was performed with open-door laminoplasty without related-complications. But he relieved some of the preoperative symptoms whose physical activities were significantly limited with the JOA scores improving from 7.5 pre-operation to 11.8 post-operation

At 1-year follow-up, 18 (9.3%) patients were in dissatisfied group, the symptom recurrence and JOA score had significant statistical differences between the two groups. No significant differences were found between the two groups in other items (Table 5). At the last follow-up, 14 (7.2%) patients were in dissatisfied group. The JOA score and symptom recurrence still had significant statistical differences. Further analysis, the JOA score of the two groups increased gradually with prolonging of restoration years but compared with dissatisfied group, the JOA scores were obviously better in satisfied group at the last follow-up (Fig. 3). No significant differences were found between the two groups in other items (Table 6).

**Table 5:** The related risk factors of satisfied and dissatisfied patients at 1-year follow-up

	Dissatisfaction (*n* = 18, 9.3%)	Satisfaction (*n* = 176, 90.7%)	*P* value
JOA scores after 1 year	12.57 ± 0.94	13.52 ± 0.96	< 0.001**
VAS neck after 1 year	2.28 ± 1.32	2.01 ± 1.28	0.393
Complications			
Axial pain (yes/no)	4/14	20/156	0.183
Symptom recurrence (yes/no)	2/16	8/168	0.023*
Postoperative depression (yes/no)	2/16	5/171	0.073

*The difference possessing statistical significance

*P < 0.05, **P = 0.000

**Table 6:** The related risk factors of satisfied and dissatisfied patients at 2-year follow-up

	Dissatisfaction (*n* = 14, 7.2%)	Satisfaction (*n* = 180, 92.8%)	*P* value
JOA scores after 2 years	4.48 ± 0.75	3.78 ± 0.73	< 0.001**
VAS neck after 2 years	11.7 ± 1.1	12.6 ± 1.0	0.001
Complications			
Axial pain (yes/no)	2/12	12/168	0.289
Symptom recurrence (yes/no)	6/8	13/167	< 0.001**
Postoperative depression (yes/no)	1/13	4/176	0.236
Others (yes/no)	1/13	9/171	0.727

*The difference possessing statistical significance

*P < 0.05, **P = 0.000

## Discussion

OPLL, a kind of senile disease, can be seen in 50–60 years old. The total incidence rate in the population is about 6.3% (8.3% in men and 3.4% in women), and up to 20% of elderly people over the age of 60 [10]. The exact cause of OPLL is still not very clear, as well as the general routine laboratory tests, such as blood routine, and blood serum protein was in normal range. Anterior decompression and interbody fusion is used to reconstruct the stability of the cervical spine to avoid further progression of ossification but accompanied by higher technical requirements and greater risk of nerve injury [11, 12]. Compared with anterior decompression surgery, the open-door laminoplasty for reducing the pressure of the injured spinal cord by indirect decompression may effectively reduce nerve injury and obtain better outcomes [13–15]. The application of open-door laminoplasty surgery is definitely indicated for patients suffering severe myelopathy on account that the pathology of OPLL is likely to cause limb dysfunction and even paralysis. Most of the patients satisfied with the results of surgery and willing to experience the procedure again. While there were 42 (21.6%) patients that are still not comfortable with their short-term outcomes and 14 (7.2%) patients with their long-term outcomes according to the PSI evaluation reminding that appropriate surgical indications and surgical timing associated with myelopathy symptoms remain to be clearly identified.

Fujimori et al. [16] reported that 20% of patients accepting surgery due to multilevel compression by OPLL were dissatisfied with the outcomes. However, this study presented a total degree of dissatisfaction without specifying posterior or anterior procedures. An advantage of our study over previous reports gained by more detailed follow-up from approximately 194 patients accepting laminoplasty procedures as the research sample. The results of our study indicated that neck pain might be associated with patient dissatisfaction in the early and middle recovery. Patient dissatisfaction at the long-term treatment outcome might be associated with the low improvement rate of JOA score and symptom recurrence.

We found that a higher hospitalization cost revealed significant influence on the dissatisfaction at discharge. The patients with a higher hospitalization cost tended to get more treatment such as more drugs promoting neurological recovery, prevention of complications, a period of professional rehabilitation training, which could lead to additional costs of hospitalization. The people usually received lower income and education, which might have some relevance to the disease-cognitive level of a person. Wang [17] et al. reported that the satisfactory degree of rural patients was worse than that of urban patients during the study of cervical spondylotic radiculopathy, but we did not have the same results in our study. Due to the low level of education, these patients, limited ability to judge the authenticity of the information, often will be vulnerable to the impact of non-formal resources of medical information. Some agencies often tend to exaggerate the actual effectiveness and even provide false information in order to maximize the benefits which could lead to great confusion and inducibility. Wholesale blindly accept, not a wise choice may mislead themselves and cast doubt on the doctor’s treatment. In addition, there is increasing consensus of the importance of economic pressure in determining a patient’s satisfaction with the results of spinal surgery. The importance of realistic expectations of surgery for improving patients’ satisfaction with outcomes has gradually been recognized [18–20]. Compared with the high-income group, the patients from low-income families often had much more optimistic expectations about their likely pain and recovery level postoperative, although the patients had been informed in detail, not only given verbally but also in written form, about the likely course of sequelae after surgery by their treating surgeon. Regardless of the severity of their illness, what they want is the result that will keep them continue to work, rather than theoretical knowledge in writing. Cost-induced dissatisfaction gradually decreases over time and is eventually accepted 6 months later.

As part of our assessment of factors for dissatisfaction, we found that axial pain as a postoperative complication for another significant adverse factor. Axial neck pain has been recognized as one of the most important complications after cervical surgery. The incidence of axial pain in individuals with posterior cervical decompression is reportedly as high as 60–80% according to previous articles [21]. Kawaguchi Y et al. reported that postoperative axial pain for posterior cervical decompression was associated with the destruction of posterior cervical muscle complex and abnormal cervical curvature [22]. In our study, the effect of axial pain on postoperative dissatisfaction is mainly manifested in the early and middle postoperative period. Axial pain for months after operation severely affects the patient’s postoperative experience and effect reached its peak in 6 months. With the relief of axial symptoms, it is no longer the main cause of long-term dissatisfaction.

Patient dissatisfaction at the long-term treatment outcome might be associated with the low improvement rate of JOA score and symptom recurrence. It is easy to understand that reasonable lower neurological functional improvement rates reflecting the more severe spinal cord injury and the poorer recovery ability had forced patients to continue to suffer pain and dysfunction, which brought the physical and mental impairments to the patients and even increased the risk of reoperation so that the patients are very painful and difficult to accept. Wang L et al. reported that the mean JOA score was 10.3 preoperatively and 15.2 at the final follow-up and the mean recovery rate (RR) was 70.7% [23]. Our research had similar results that JOA scores were significantly higher at the last follow-up than that before operation. The reason why JOA scores do not significantly affect dissatisfaction at the early and middle stages may be that as the symptoms improve gradually, it brings hope to the patients. However, with no apparent remission of symptoms after 1 year, the impact of low improvement rate of JOA score on life is gradually coming out. Another adverse factor on patient dissatisfaction at the long-term is symptom recurrence, which brought the physical and mental impairments to the patients so that the patients are very painful and difficult to accept. Ha Y et al. reported that growth of ossification of the posterior longitudinal ligament (50%) was the primary cause of revision surgery and noted that clinical outcomes of revision surgery are similar to the outcomes of patients who did not require revision surgery [24]. So that many of the patients do not have the courage to accept the second operation. In the previous studies, age and duration of symptoms reported could influence the outcome of surgical treatment [25]. Kim et al. reported older age had a negative effect on account of neuropathies and vascular insufficiency [26]. However, these factors have never been substantiated in our study. Six patients were found cerebrospinal fluid leakage for posterior longitudinal ligament adhered to the dura mater [27–30], which had no statistical difference for the long-term outcomes between the two groups. The complications affected the patient’s mood and surgical experience and also added to the cost of treatment which might lead to dissatisfaction. Therefore, the maximum reduction of postoperative complications can increase the good experience and satisfaction for patients.

This study is associated with several limitations. First, patients satisfaction is believed to be not only associated with many variables but also an attitudinal response to value judgments coming from their clinical experience. Different people may have different subjective feelings about the same thing because of the different environment. For finite condition, objective postoperative impact test was unable to get. Second, due to the limited capacity of expression, part of the questionnaire is completed by the patient’s family, rather than the patient himself. Families tend to be closer to their own subjective understanding, and sometimes cannot precisely reflect the true idea of the patients. In addition, the PSI evaluating outcomes of surgery are easy to use but quite simple without detailed distinction. Despite these limitations, we believe that this study has important guiding information in clinical work. We think the postoperative change of the ossification is very meaningful clinical indicator, but because it was a retrospective study, no routine imaging review was performed after cervical spine surgery. We hope that could be elaborated in future research.

## Conclusions

In conclusion, to patients undergoing open-door laminoplasty for cervical OPLL, hospitalization cost and neck pain might be mainly associated with patient dissatisfaction at the early and middle recovery. Patient dissatisfaction at the long-term treatment outcome might be mainly associated with the low improvement rate of JOA score and symptom recurrence.

## Data Availability

The datasets generated and analyzed during the current study are available from the corresponding author on reasonable request.

## Abbreviations

BMI: Body mass index
IHSI: Intramedullary high-signal intensity
JOA: Japanese Orthopaedic Association
OPLL: Ossification of posterior longitudinal ligament
PSI: Patient Satisfaction Index
VAS: Visual analog scale
